# Atypical cutaneous presentation of AOSD with persistent itchy urticaria: A case report

**DOI:** 10.1097/MD.0000000000036251

**Published:** 2023-12-15

**Authors:** Jingfeng Lou, Xingping Zhang

**Affiliations:** a Department of General Medicine, Chengdu Second People’s Hospital, Chengdu, China.

**Keywords:** adult-onset still's disease, atypical, case report, skin rash

## Abstract

**Rationale:**

Adult-onset Still’s disease (AOSD) is a rare multisystem disorder considered a complex autoinflammatory syndrome. The clinical and biological features of AOSD typically include a high fever with arthritic symptoms, evanescent skin rash, sore throat, striking neutrophilic leukocytosis, hyperferritinemia, and abnormal liver function. The typical rash and fever are important diagnostic clues for AOSD. Here, we report a case of atypical rash manifesting as persistent itchy urticaria.

**Patient concerns:**

A 57-year-old female presented with a 6-day history of fever. During her hospital stay, she progressively developed rashes that were not associated with fever, primarily distributed on her back and the distal extremities, and associated with pronounced itching. The rash was initially suspected to be urticaria; however, the patient exhibited a poor response to antihistamines. After malignancies and other rheumatic diseases were excluded, the diagnosis leaned towards AOSD based on diagnostic criteria. The patient’s fever was well controlled with the initiation of glucocorticoids, and no further rashes were observed.

**Diagnoses:**

Although the patient exhibited atypical rashes, after ruling out malignancies and other rheumatic diseases, she met 2 major and 3 minor criteria. Based on Yamaguchi’s criteria, the patient was diagnosed with AOSD.

**Interventions:**

Initially, the patient was administered an intravenous infusion of methylprednisolone at 40 mg once daily. This was later transitioned to oral administration with gradual dose reduction.

**Outcomes:**

Follow-up at 1 year showed no recurrence of the rash, with a stable condition and no relapse.

**Lessons:**

This case provides valuable insights for the early diagnosis of AOSD, emphasizing the importance of considering this diagnosis even when presenting with atypical skin rash.

## 1. Introduction

Adult-onset Still’s disease (AOSD), first described in 1971 is a rare, idiopathic, multisystem inflammatory disorder.^[1]^ Existing epidemiological studies report the incidence of AOSD between 0.16 to 0.4 per 100,000 people and estimated prevalence between 1 to 34 cases per 1 million people.^[2,3]^ AOSD is mainly characterized by fever, joint pain or arthritis, sore throat, swollen lymph nodes and evanescent skin rash.^[4,5]^ Several sets of classification criteria have been proposed for research and diagnosis, all of which stem from retrospective studies. Currently, the majority of studies on AOSD involve patients who meet the Yamaguchi and/or Fautrel criteria. The etiology of AOSD is unknown, and the range of differential diagnosis is wide, making the diagnosis complex, especially when early diagnosis is difficult.

Although early diagnosis of AOSD is difficult and it does not have specific laboratory markers, such as many diseases, the typical salmon-pink maculopapular rash is an important characteristic for making a definitive diagnosis.^[6,7]^ This typical rash is usually asymptomatic, appearing with fever and disappearing as the fever subside. The typical rash is a very important part of the major diagnostic criteria, and other types of rashes are not included in the diagnostic criteria, so they are not considered to have diagnostic significance. However, recent studies have increasingly reported atypical rashes in AOSD patients.^[8–14]^ We report a case of an AOSD patient with an atypical rash, which was not related to fever, persisted for a long time, and manifested as pronounced itchy urticaria.

## 2. Case presentation

A 57-year-old woman visited Chengdu Second People’s Hospital for medical care, presenting with a 6-day history of fever characterized by chills and fatigue. The patient’s temperature increased to 39 °C. Despite initial treatments with cephalosporin and ibuprofen, she persistently experienced recurrent fevers peaking at approximately 39 °C, most commonly in the evenings or at night. No notable details were reported in her personal or family medical history.

**Figure 1.**
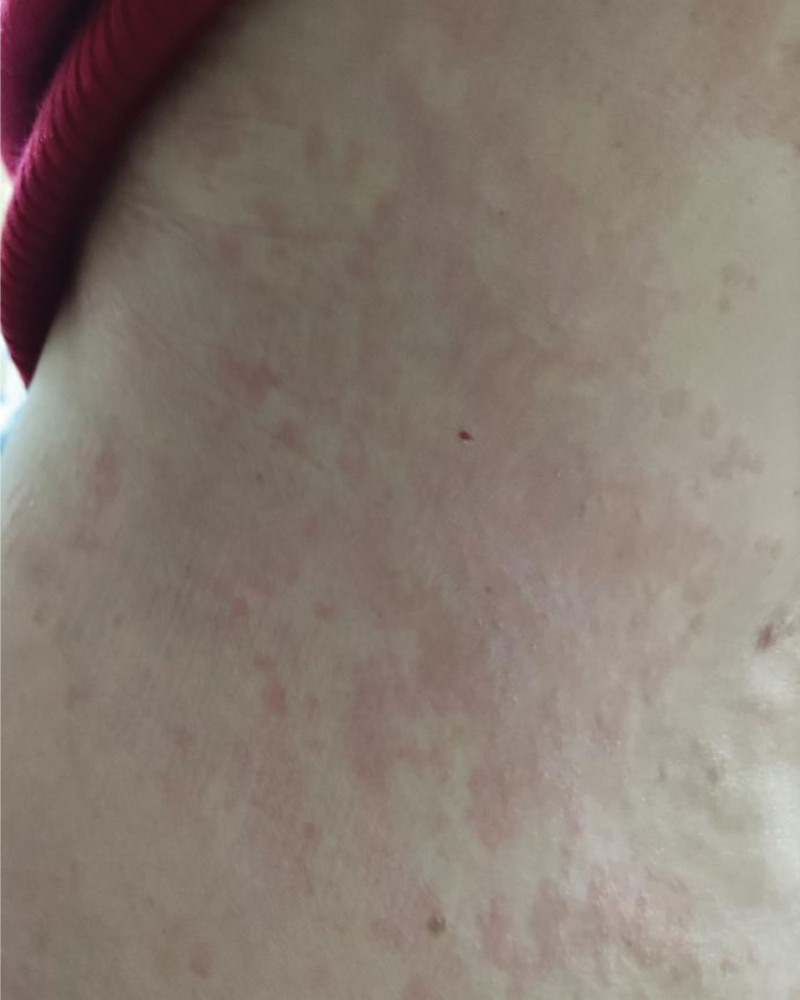
Urticarial eruption on the back, with some presenting as erythema nodosum.

**Figure 2.**
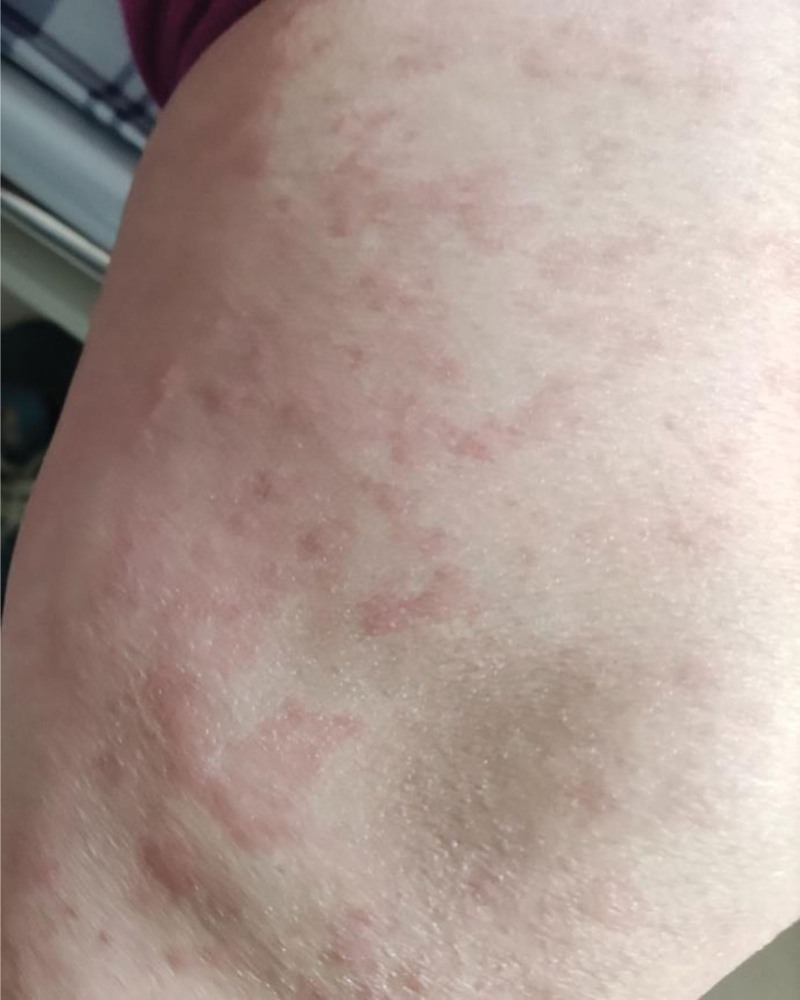
Urticarial patches on the side of the knee.

**Figure 3.**
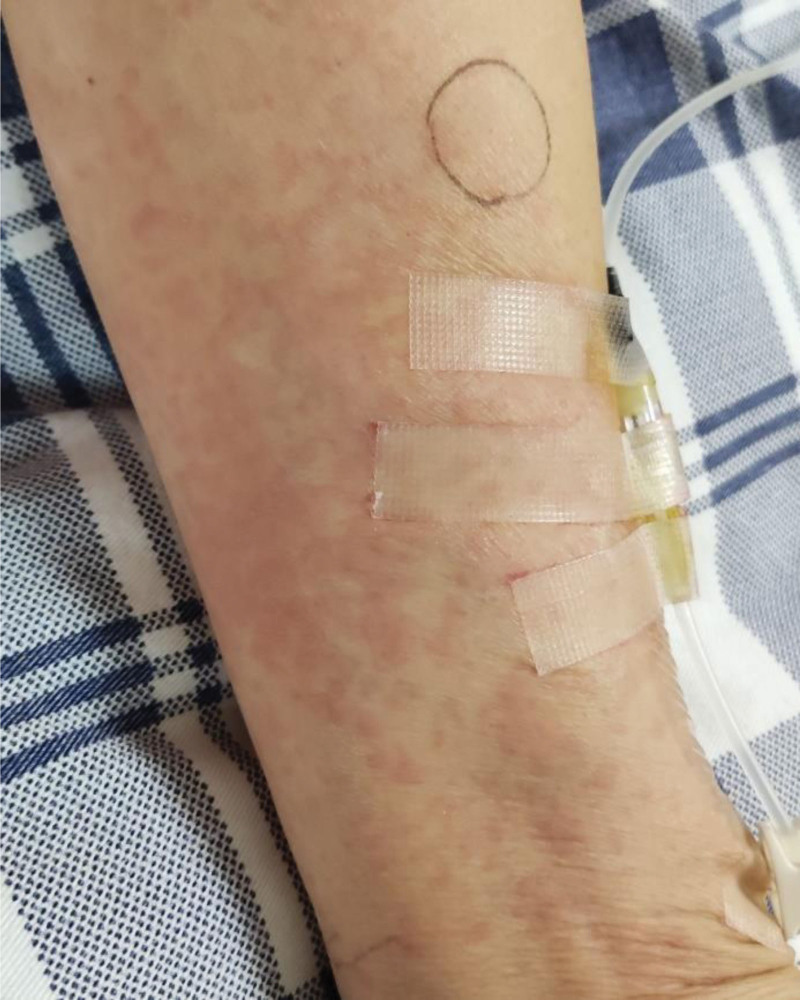
Plaques on the distal upper limbs.

**Figure 4.**
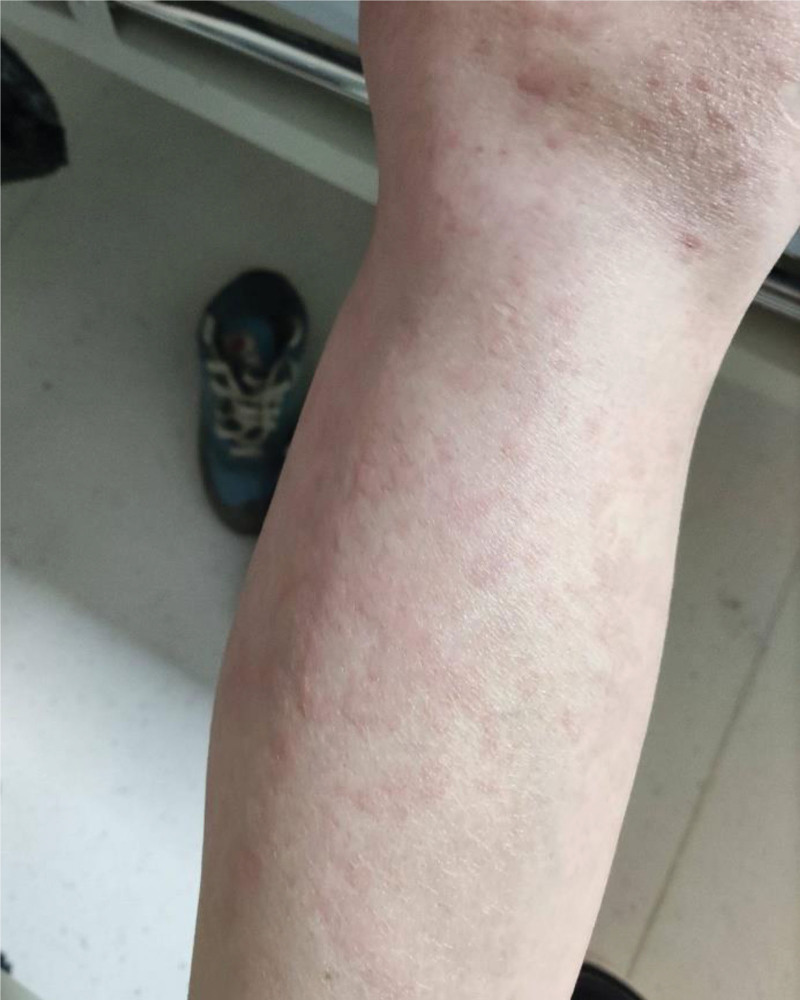
Plaques on the distal lower limbs.

At the time of admission, physical examination of the patient showed no significant abnormalities and no rash. However, on the second day of hospitalization, she developed an urticaria rash on her lower legs, accompanied by pronounced itching. The rash appeared to have no clear correlation with the fever. We initially suspected acute urticaria and administered ebastine as an anti-allergic treatment; however, there was no notable improvement. Subsequently, the rash gradually expanded in coverage, manifesting on her back and distal parts of her limbs, with pronounced itching (Figs. 1–4). We administered cetirizine and calcium gluconate, but observed no notable improvement. The rash persisted for an extended duration and showed no clear correlation with the fever.

Additionally, laboratory results indicated polymorphonuclear leukocytosis, with a white blood cell count of 20.0 × 10^9^/L, predominantly composed of 94.6% neutrophils. Inflammatory markers were significantly elevated, with high-sensitivity C-reactive protein levels dramatically elevated at 92.85 mg/L, and procalcitonin levels heightened to 2.90 ng/mL. Coagulation markers such as D-dimer and fibrin degradation products were also increased, registering at 27.89 and 80.30 μg/mL, respectively. Furthermore, the patient’s ferritin level was markedly elevated beyond 2000 ng/mL, and the lactate dehydrogenase level raised to 329 U/L. Additionally, serology revealed increased titers of hCMV-IgG (107.0 U/mL), Rubella IgG (41.3 IU/mL), and HSV-IgG (>30.0 index). Her EBV-VCA-IgG titer was significantly increased, surpassing 750.0 U/mL. A comprehensive battery of tests was conducted, including liver and renal function, HIV, tumor markers, galactomannan and (1-3)-beta-D-glucan tests, tuberculin skin test, tuberculosis antibody, Mycoplasma pneumoniae, Chlamydia pneumoniae, antineutrophil cytoplasmic antibodies, markers for hepatitis B and C, HBV DNA, blood culture, and urine culture. All the tests yielded negative results. Computed tomography of the cranium, chest, abdomen, and pelvis revealed no significant abnormalities. Likewise, no anomalies were discerned on thyroid ultrasound.

Considering the patient’s fever and elevated infection indicators, infection could not be ruled out. After admission, the patient was started on cefoperazone/sulbactam 1.5/1.5 g for infection control. owing to her persistent fever and elevated infection indicators, moxifloxacin (0.4 g) was additionally administered. However, the patient continued to have recurring fever, and the rash did not subside. Subsequently, the patient underwent further auxiliary examination. Lymph node ultrasound identified enlargement of the right axillary lymph nodes without obvious structural abnormalities. Bone marrow aspiration revealed hypercellularity; however, bone marrow hyperplasia was normal, with no evidence of acute leukemia, non-Hodgkin ‘s lymphoma, or myelodysplastic syndromes.

After excluding malignancies and rheumatic diseases, the patient met two of the major criteria, fever and elevated white blood cell count, as well as two of the minor criteria, lymphadenopathy and negative results for both rheumatoid factor and antinuclear antibody. Although the patient displayed a rash, it presented with persistent urticaria with pronounced itching and showed no clear correlation with fever; the distribution of the rash also differed from that of a typical one. Hence, with only 4 criteria met, a definitive diagnosis of AOSD has not yet been established. A few days later, her liver function was found to be abnormal. The diagnosis of AOSD was made when more than five of Yamaguchi’s criteria were fulfilled. Intravenous injection of meprednisone was initiated at a dose of 40 mg daily. The next day, her temperature returned to normal, and by the third day, the rash had completely disappeared. The patient continued to take oral methylprednisolone 32 mg after discharge. Over the next 6 months, the dose of methylprednisolone was gradually reduced. Follow-up at 1 year showed no recurrence of the rash, with a stable condition, and no relapse. The patient did not experience any adverse drug reactions during treatment.

## 3. Discussion

AOSD is a rare, multisystem inflammatory disease with unknown etiology. The primary clinical manifestations include fever, arthritis, rash concurrent with fever, sore throat, lymphadenopathy, and hepatosplenomegaly. The diagnosis of AOSD mainly depends on the Yamaguchi standard or Fautrel standard,^[6,15]^ which both mention that it is necessary to exclude infection, tumor, blood system diseases and other diseases before diagnosis. Early detection is challenging and prone to misdiagnosis.

The characteristic rash of AOSD manifests as transient salmon-pink macules. Typical rash is a crucial symptom in the diagnosis of AOSD. However, the patient in this case presented with a persistent itchy urticaria-like rash, the appearance of which did not align with the fever. Furthermore, the distribution of the rash on the trunk and distal extremities was atypical compared with the characteristic rash of AOSD.

In recent years, some case reports have documented atypical rashes. Some studies have described rash as persistent plaque. Lee et al^[11]^ summarized 36 cases diagnosed with AOSD in a hospital, of which 28 presented with persistent rashes. And in addition to being persistent, atypical rashes can manifest as various types. Some present as urticarial-like rashes. Affleck et al^[9]^ reported 1 case of AOSD presenting as migratory pruritic urticarial rash. Red and urticated plaques were initially distributed in both legs. After 5 months, the rash became erythematous, fixed, and plaque-like, affecting the arms, dorsal hands, upper chest, and back. Akkurt et al^[16]^ reported 5 cases of AOSD. Some patients presented with edematous papular eruptions, whereas others displayed widespread erythematous pigmented plaques with a linear pattern. Criado et al^[17]^ reported a patient with erythematous edematous plaque.

In this patient’s auxiliary examination, both C-reactive protein and ferritin levels were significantly elevated, with the increase in ferritin level being particularly pronounced. Elevated C-reactive protein and ferritin levels are common in patients with AOSD. Previous studies have shown that ferritin levels in AOSD patients are higher than those in patients with other autoimmune diseases, inflammatory diseases, infectious diseases, or neoplastic diseases. An increase in ferritin level exceeding 5 times the normal value strongly suggests AOSD.^[18,19]^ Additionally, joint pain is a common symptom in patients with AOSD and is a crucial clue in diagnosing AOSD.^[20]^ However, our patient did not exhibit arthralgia throughout the course of the disease, indicating that the symptoms of AOSD are nonspecific.

Therefore, we cannot consider AOSD only when a typical rash appears, as there have been many cases of patients presenting with atypical rashes. When we encounter patients with fever, accompanying rashes, and symptoms such as joint pain, we should be highly vigilant for AOSD. However, we should concurrently exclude malignant diseases, such as hematological disorders. The presence of atypical skin features could be of significant importance in diagnosing AOSD and might be integrated into the diagnostic criteria.

## Author contributions

**Conceptualization:** Jingfeng Lou, Xingping Zhang.

**Data curation:** Jingfeng Lou.

**Supervision:** Xingping Zhang.

**Writing – original draft:** Jingfeng Lou.

**Writing – review & editing:** Jingfeng Lou.
